# Engaging adolescents with HIV to ensure better health and more informed research

**DOI:** 10.7448/IAS.20.4.21569

**Published:** 2017-05-16

**Authors:** Carlo A Oliveras Rodriguez

**Affiliations:** ^a^ Adolescent HIV Treatment Coalition, International AIDS Society, Geneva, Switzerland

**Keywords:** Adolescent, HIV, engagement, youth, investment, global, development, AIDS.

​​​There have been previous efforts to highlight the unmet needs of adolescents living with HIV (ALHIV). Examples include reports on the Romanian children who had been infected through contaminated needles and blood transfusions and were entering adolescence in the mid-2000s with little hope for education, treatment, and a secure future [1], and on the Kenyan children who were not being included in national surveillance surveys and faced social and policy barriers to access testing and treatment [2]. While these seldom led to systematic change, data on rising and alarmingly high mortality rates have galvanized advocates and policy makers to refocus on youth [3,4]. Between 2005 and 2012, the number of AIDS-related deaths decreased by 30% for all ages except among adolescents, who experienced a 50% increase in that same period [5]. Moreover, two-thirds of new HIV infections in 2012 occurred among youth aged 15–24 [5]. There are numerous and complex reasons behind our failure to prevent these deaths, and each needs a dedicated response in order to improve the health outcomes of ALHIV.

In recent years, discussions on the health of ALHIV have been centre stage at many national and international forums, and have called for a revitalized community-wide response to support ALHIV. This requires a better understanding of the real-world situations faced by adolescents who have grown up with HIV as well as those recently infected, across diverse contexts and as members of communities identified as “most at risk.” Adolescents who use drugs, who sell sex, and who are lesbian/gay/bisexual/transgender are less likely to be served by traditional HIV care programmes. The need to support optimal interventions to help them thrive, and the meaningful engagement of ALHIV throughout the response is key to support them to the next life stage.

The essential part of any solution to these challenges is the engagement of young people living with HIV (YPLHIV) in their own care, and the development of policies and research that affect them. Currently, efforts to make ALHIV a part of the HIV response are mostly on a voluntary basis, and may not be flexibly organized around their available evening and weekend schedules. Whether they are asked to be peer supporters or attend the occasional policy meeting, this can pull them away from their education or employment during a time in their lives when they need to be building social and economic capital for themselves. Unlike older adults, they have yet to have established vocations and stable employment. In order to ensure stronger youth engagement, financing for broader and consistent participation during this period of life is needed. Moreover, because ALHIV are young, the collective view may have that they have less expertise to offer, and thus their time is somehow less valuable. We need to strike a better balance between promoting participation and understanding that volunteering should not be a permanent arrangement for youth. Some positive examples of enhanced participation and engagement are seen in the development of networks of YPLHIV and young key populations [6,7]. Investments into fostering leadership development among youth that provide them with tools to develop professionally and academically are key for a revitalized youth response.

Adequate treatment literacy and the right to make decisions about one’s own health are central to empowering ALHIV to be able to engage in decisions about their own care. Finding innovative ways to establish youth as actors in their own treatment decision-making would have onward benefits as they gain confidence navigating healthcare systems, and would promote adherence and retention. However, restrictive consent laws or other practices that limit access to healthcare and comprehensive sexual and reproductive health education uniquely and negatively impact ALHIV. While much work has been done around consent procedures with regards to the use of human subjects in research, more efforts are needed around improving consent policies for adolescent clinical care. The Convention of the Rights of the Child, which defines a child as being under the age of 18, unless the age of majority is otherwise defined by national legislation, in Article 12 expresses that “*States Parties shall assure to the child who is capable of forming his or her own views the right to express those views freely in all matters affecting the child, the views of the child being given due weight in accordance with the age and maturity of the child*” [8]. Nevertheless, national governments frequently opt not to adjust the age of consent for adolescents to receive medical care or participate in research without the knowledge of parents or guardians, creating substantial barriers to testing for HIV and other sexual transmitted infections, and reproductive healthcare, as well as to research participation.

In order for research aiming to improve the lives of ALHIV to translate into programmatic impact, youth should be involved from the very beginning of a study [9]. Protocols, methodologies, and community advisory activities need to take into consideration their views. The language used in these documents should be made both culturally appropriate and better suited to the developmental stage of the adolescent target population. When youth are not engaged to provide feedback on expectations for recruitment, enrolment, and retention, it is unreasonable for researchers to expect their participation will be the same as for older adults. However, variable participation should also not lead to the impression that youth are unreliable, but that research processes need to be more youth friendly. Successful examples of youth engagement in research have demonstrated that enabling multiple modes of participation that take into consideration developmental stage and social factors is possible [9]. Establishing ways for clinicians and researchers to teach ALHIV about the importance of research and interpret its outcomes to inform them about their own health can strengthen the scope and depth of the advocacy that is needed to improve the health outcomes of ALHIV.

Engaging ALHIV in the broader HIV response and in research specifically is not an easy task, yet its benefits are far greater than its challenges. The responsibility for facilitating this effort lies with all stakeholders, whether from within civil society, among implementers, or across funders. With guidance and mentorship, they can become more aware and empowered regarding their own health and rights to access care and participate (or not participate) in research. Supporting ALHIV in achieving equity in care and improve treatment outcomes requires all of us to invest in their individual and collective development.

## List of abbreviations

ALHIV, Adolescents living with HIV; ERB, Ethical review boards; NGO, Non Governmental Organizations; UN, United Nations; YPLHIV, Young people living with HIV.
